# Mesenteric Cyst: A Case Report of a Healthy, Five-Year-Old Patient

**DOI:** 10.7759/cureus.34443

**Published:** 2023-01-31

**Authors:** Pedro Sureda, Rafael Mena Canto, Carmen L Familia Sanchez

**Affiliations:** 1 Pediatric Surgery, Torre Profesional Corazones Unidos, Santo Domingo, DOM; 2 Pediatrics and Neonatology, Centro de Obstetricia y Ginecologia, Santo Domingo, DOM

**Keywords:** pediatrics, surgery, mesentery, cyst, abdominal mass

## Abstract

Mesenteric and omental cysts can be seen at any age, with one in three cases seen in patients under 15 years old. These cysts represent one in 20,000 pediatric admissions. Here we share the case of a five-year-old female patient at a health center in a developing country, with the purpose of contributing to documentation in the region.

## Introduction

Mesenteric and omental cysts are cystic abdominal swellings present with painless, often uniform enlargement of the abdomen. They can occur at any age, but one in three cases is seen in patients younger than 15 years old [1,2].

The reported incidence of mesenteric cysts is one in 140,000 hospital admissions and one in 20,000 pediatric admissions [3-5]. About 60% of pediatric patients with mesenteric cysts are males [6].

## Case presentation

**Figure 1:**
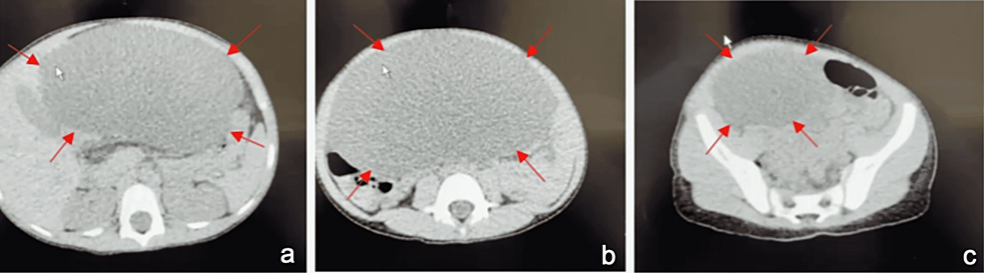
Non-contrast CT of the abdomen. Images a, b, and c show different sections of the cyst. Red arrows mark mesenteric cyst.

An otherwise healthy five-year-old female patient presented to the Emergency Department with a history of crampy abdominal pain and vomiting for 12 hours of duration. The patient did not have any relevant personal or family history. On physical examination, a distended abdomen and remarkable tenderness to palpation were noticed. An abdominal ultrasound revealed a cyst in the abdominal cavity, which needed a computerized axial tomography (Figure 1) for further evaluation. 

**Figure 2:**
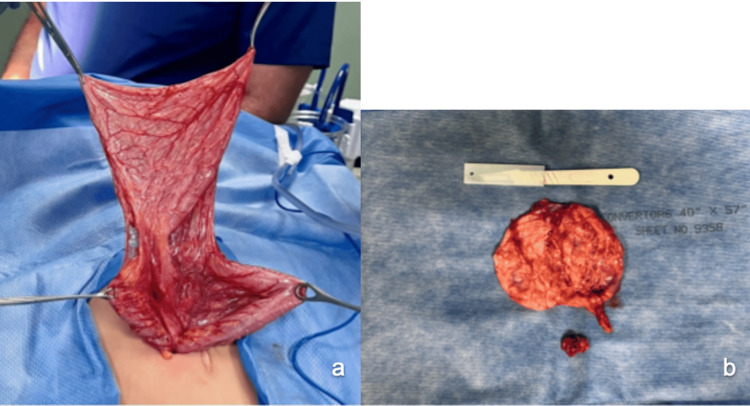
Mesenteric cyst. a. Drained mesenteric cyst. b. Resected mesenteric cyst.

The patient was taken to the operating room the next day with the diagnosis of cystic abdominal mass/probable mesenteric cyst. An exploratory laparotomy was performed, finding a cystic mass in the transverse mesocolon from which 1,700 mL of serous content was aspirated and the cyst was completely resected (Figure 2). The peritoneal fluid analysis revealed cloudy fluid as the only abnormality.

The recovery was uneventful and the patient was discharged on the second postoperative day in good general condition. There were no complications in the follow-up appointment the next month. The alpha-fetoprotein results were less than 1.50 mg/dL and the histopathological report confirmed the diagnosis of a simple mesothelial-type mesenteric cyst without histological signs of malignancy.

## Discussion

Although there are many theories about the cause of the formation of mesenteric and omental cysts, there is no consensus on its etiology. The most popular theory, proposed by Gross, is a benign proliferation of lymphatics in the mesentery that do not communicate with the lymphatic drainage system, analogous to lymphatic malformations of the neck [7].

Malignancy has been reported in samples from up to 3% of adult patients [8]. There are no reports of associated malignancy in children, where these tumors are considered benign and the risk of recurrence is minimal [9].

In adults, most mesenteric and omental cysts are found incidentally at the time of an abdominal examination [10]. On the other hand, in the pediatric population, it can present as acute abdominal pain, vomiting, and bloating [11].

On physical examination, the diagnosis of a mesenteric or omental cyst is usually distinguished from other intra-abdominal cystic lesions by the presence of a palpable and freely mobile mass. Extremely large lesions can fill much of the abdominal cavity and feel less mobile, making the diagnosis difficult. Unless they are very large, ovarian and common bile duct cysts are difficult to palpate due to their location in the pelvis and right upper quadrant, respectively. Gastrointestinal duplication cysts may have a similar finding on physical examination when arising from the jejunum and ileum, as these organs are not attached to the retroperitoneum [12,13].

Ultrasound is the most common modality used to evaluate mesenteric and omental cystic lesions. It is relatively inexpensive, non-invasive, and can be repeated to track injuries over time. Alternative studies include magnetic resonance imaging (MRI) and computed tomography (CT). If the diagnosis is unclear and the physician is trying to decide between a conservative approach and a surgical intervention, these imaging modalities can help distinguish mesenteric and omental cysts from other intra-abdominal cysts. Abdominal CT helps to characterize the size more precisely, delimits the extent of the mass, shows the relationship with other abdominal organs, and helps detect calcium deposits, as well as can help detect compression of the adjacent structures and whether there is a retroperitoneal extension [3,14].

Given the difficulty in distinguishing between these lesions on ultrasound, most patients will need a laparoscopy to confirm the diagnosis. This can occur in the event of cyst torsion, as it is likely to mimic a condition requiring urgent exploration (e.g., ovarian torsion or acute appendicitis). In cases where the patient is not a good candidate for surgery, additional imaging with CT or MRI can help limit the differential diagnosis [2,3,15]. The differential diagnosis for mesenteric cyst includes gastrointestinal duplication cyst, ovarian cyst, paratubal cyst, choledochal cyst, pancreatic cyst, splenic cyst, renal cyst, adrenal cyst, omental cyst, omphalomesenteric duct cyst, urachal cyst, hydatid cyst, hydronephrosis, and ascites [4].

Mesenteric cysts can be found anywhere in the mesentery of the small intestine, large intestine, or retroperitoneum. They are most frequent along the mesentery of the small intestine (60%), followed by the large intestine (24%) and the retroperitoneum (15%) [1].

The treatment of omental and mesenteric cysts depends on their presentation. These presentations range from an incidental radiographic or intraoperative finding to a serious and life-threatening finding secondary to volvulus or intestinal obstruction. The diagnosis is usually made in the setting of acute abdominal pain, and treatment is usually surgical resection [1,4,5].

In cases where the cyst affects a significant part of the intestine or extends to a large part of the retroperitoneum, partial resection with marsupialization and the use of sclerotherapy agents have been described. The goal of the intervention is to eliminate the source of the symptoms, preferably by complete excision. Due to the high recurrence rate of drainage alone, the cyst lining must be sclerosed with agents such as 10% glucose or ethyl alcohol [4].

Approximately 40% of cysts are discovered incidentally at laparotomy for other conditions and must be removed if the outcome of the primary surgery is not negatively affected. Cysts that are discovered on abdominal pain imaging may be the cause of the symptom and should be removed as soon as possible [5].

Observation alone can result in twisting of the cyst, intestinal obstruction, volvulus, or bleeding in the cyst that can mimic an acute abdomen. Moreover, cysts can get infected or might rupture. Although rare, a malignant transformation has been reported; therefore, it is not advisable to keep a cyst unresected [3].

Complete excision of the cyst is curative. However, because of the multiloculated nature of some of the cysts, intraoperative rupture can lead to incomplete resection and thus recurrence. Some small cysts may also be missed, which could become enlarged and cause a recurrence. The recurrence rate is generally low (0-15%) and is even lower after complete surgical resection [2,4].

## Conclusions

In pediatric patients, mesenteric cysts can present with abdominal pain, vomiting, and bloating, as seen in this case. Ultrasound is commonly used due to availability and non-invasiveness, but abdominal CT delimits the extent of the mass more precisely. It is not normally associated with malignancy in children and the goal of the intervention is to improve the symptoms. Complete excision of the cyst can be curative with a low recurrence rate.
